# Immobilization of Platelet-Rich Plasma onto COOH Plasma-Coated PCL Nanofibers Boost Viability and Proliferation of Human Mesenchymal Stem Cells

**DOI:** 10.3390/polym9120736

**Published:** 2017-12-20

**Authors:** Anastasiya Solovieva, Svetlana Miroshnichenko, Andrey Kovalskii, Elizaveta Permyakova, Zakhar Popov, Eva Dvořáková, Philip Kiryukhantsev-Korneev, Aleksei Obrosov, Josef Polčak, Lenka Zajíčková, Dmitry V. Shtansky, Anton Manakhov

**Affiliations:** 1 Scientific Institute of Clinical and Experimental Lymphology-Branch of the ICG SB RAS, 2 Timakova str., 630060 Novosibirsk, Russia; solovevaao@gmail.com (A.S.); svmiro@yandex.ru (S.M.); 2 National University of Science and Technology “MISiS”, Leninsky pr. 4, 119049 Moscow, Russia; andreykovalskii@gmail.com (A.K.); permyakova.elizaveta@gmail.com (E.P.); zipcool@bk.ru (Z.P.); kiruhancev-korneev@yandex.ru (P.K.-K.), shtansky@shs.misis.ru (D.V.S.); 3 Research Institute of Biochemistry, 2 Timakova str., 630117 Novosibirsk, Russia; 4 RG Plasma Technologies, CEITEC–Central European Institute of Technology, Masaryk University, Purkyňova 123, 61200 Brno, Czech Republic; evke.kedronova@gmail.com (E.D.); lenkaz@physics.muni.cz (L.Z.); 5 Chair of Physical Metallurgy and Materials Technology, Brandenburg Technical University, 03046 Cottbus, Germany; aleksei.obrosov@b-tu.de; 6 CEITEC-Central European Institute of Technology, Brno University of Technology, Technická 3058/10, 61600 Brno, Czech Republic; polcak@fme.vutbr.cz; 7 Institute of Physical Engineering, Brno University of Technology, Technicka 2896/2, 616 69 Brno, Czech Republic

**Keywords:** polycaprolactone, nanofibers, COOH plasma, platelet-rich plasma, cell viability, PRP immobilization

## Abstract

The scaffolds made of polycaprolactone (PCL) are actively employed in different areas of biology and medicine, especially in tissue engineering. However, the usage of unmodified PCL is significantly restricted by the hydrophobicity of its surface, due to the fact that its inert surface hinders the adhesion of cells and the cell interactions on PCL surface. In this work, the surface of PCL nanofibers is modified by Ar/CO_2_/C_2_H_4_ plasma depositing active COOH groups in the amount of 0.57 at % that were later used for the immobilization of platelet-rich plasma (PRP). The modification of PCL nanofibers significantly enhances the viability and proliferation (by hundred times) of human mesenchymal stem cells, and decreases apoptotic cell death to a normal level. According to X-ray photoelectron spectroscopy (XPS), after immobilization of PRP, up to 10.7 at % of nitrogen was incorporated into the nanofibers surface confirming the grafting of proteins. Active proliferation and sustaining the cell viability on nanofibers with immobilized PRP led to an average number of cells of 258 ± 12.9 and 364 ± 34.5 for nanofibers with ionic and covalent bonding of PRP, respectively. Hence, our new method for the modification of PCL nanofibers with PRP opens new possibilities for its application in tissue engineering.

## 1. Introduction

In the last few decades, attention was drawn to biodegradable polycaprolactone (PCL) nanofibers for its usage in tissue engineering due to their outstanding properties [1]. PCL is a semicrystalline, white-colored, bioresorbable polymer belonging to aliphatic polyesters. The PCL homopolymer has total degradation time of 2–4 years in the body, depending on the starting molecular weight used for the implant [2]. However, PCL as hydrophobic polyester has limited use, especially in a predominantly hydrophilic bioenvironment for tissue engineering. Its modification with hydrophilic synthetic and bio-polymers opens new horizons for its applications [3]. Compared to more expensive gelatin or collagen nanofibers, PCL has better mechanical properties, whereas if we compare PCL with Nylon 6 (a semicrystalline polyamide), the latter one has poor biodegradability. Oligomers of Nylon 6 are biodegradable, but not polymers [4]. It should be noted that this material is not sufficiently suitable for maintaining cell adhesion, proliferation, and differentiation, due to the hydrophobic nature of its surface. This drawback can be overcome by surface modification of PCL nanofibers leading to increasing biocompatibility of the material [5,6,7].

The plasma deposition of functional thin layers is a method could be chosen to enhance the biocompatibility of the nanofiber surface [8]. The functional groups (COOH, NH_2_, OH, etc.) increase the wettability and surface free energy of the nanofibers and, moreover, reactive surface groups can be used for the immobilization of biomolecules [9]. The plasma-deposited thin layers are used to immobilize antibodies, growth factors, and peptides, as this robust, environmentally friendly technique is substrate-independent and particularly efficient [10,11].

In order to use the nanofibrous mats for regenerative medicine, the substrates should enhance the functional activity of adhered cells, or attract the cells into the wounded area [12,13]. Tissue engineering aimed at replacement or restoration of damaged tissues with synthetic structures are designed to mimic the extracellular matrix (ECM). As cell adhesion and viability can be ultimately improved if the surface of nanofibers is functionalized with vital biological proteins, peptides, and growth factors. It would be beneficial to combine the plasma deposition of reactive groups on nanofibers with the robust immobilization of vital biomolecules to significantly increase the biocompatibility of the nanofibrous mats [14,15,16,17]. For instance, in order to enhance the osteogenic differentiation of mesenchymal stem cells (MSCs), PCL scaffolds were modified with fibronectin and hyaluronic acid [18,19]. Collagen and vascular epidermal growth factor (VEGF) immobilization are often used for stimulation of angiogenesis in the wounded area [20].

Recently, platelet-rich plasma (PRP) has been actively applied in the therapy of burns, acute and chronic disruptions of the musculoskeletal system, dental implants, the therapy of periodontal tissues disease [21,22], and dermatology [23]. The efficiency of PRP is related to the high concentration of bioactive molecules and growth factors. These biomolecules are released owing to the destruction of thrombocytes. The mixture of active molecules includes multiple growth factors such as epidermal growth factor, basic fibroblast-derived growth factor, vascular endothelial growth factor, insulin-like growth factor-1, platelet derived growth factor, transforming growth factor beta 1, adhesion molecules-vascular cell adhesion molecule-1, intercellular adhesion molecule-1; protease inhibitors, proteoglycans, specialized chemokines, and cytokines [24,25]. These growth factors influence the cell adhesion, proliferation, and functional activity of cells [26]. PRP also contains structural constituents of extracellular matrix (ECM) such as fibronectin, vitronectin, and thrombospondin. Furthermore, fibronectin enhances the cell adhesion and interaction between cells [27].

Although the high activity of PRP was confirmed, there are several studies reporting its short-term effect. In order to prolong the PRP activity and activation (by lyses) and PRP embedment (into the matrix structures) are often used [28].

The immobilization of PRP onto the matrix led to the retention of the PRP structure, and to the prolonging of PRP activity. As a result, the enhancement of cell proliferation and differentiation will be extended.

Liu et al. demonstrated the nanofibers with PRP incorporated by the emulsion electrospinning technique allowed to achieve the controlled and sustained release of growth factors (GFs) [29]. The enhancement of cell attachment and proliferation and intrinsic cartilage healing was demonstrated [29]. However, the emulsion electrospinning of PRP and PCL leads to the limited effect of the PRP. This obstacle could be partially solved if water-soluble polymeric nanofibers are used. Nevertheless, in this case, the obtained PRP-loaded nanofibers do not enable controlled release of biomolecules, including GFs, resulting in their immediate release into media. The most prominent effect was observed within the first 24 h of incubation [30]. Thus, the most perspective approach would be immobilization of the PRP on the surface of plasma coated PCL nanofibers. In our previous work, it was reported that electrospun PCL nanofibers could be coated with COOH and NH_2_ groups, and that these plasma coatings improve the biocompatibility of nanofibers [31]. However, the immobilization of the PRP on plasma-coated PCL nanofibers and its influence on the biocompatibility of prepared nanofibrous mats has never been tested before.

The effectiveness of tissue engineering constructions depends on the type of cell and the substrate. Many strategies employ the mesenchymal stromal cells (MSCs) as the promising candidates for cell based therapy. Numerous studies have shown the achievement of PRP for maintaining the viability and proliferation of bone marrow (BM) MSCs. The platelet lysate was prepared by PRP freeze/thaw cycles showed high efficiency of maintaining MSC functions, including high cell proliferation level [32].

In the current research, the influence of different immobilization strategies of the PRP on the surface of PCL nanofibers has been studied. It was found that PRP cannot be attached to the untreated PCL nanofibers, while the deposition of CO_2_/C_2_H_4_ plasma polymers significantly improves MSC adhesion and the immobilization efficiency of the PRP on the PCL nanofibers. Finally, the best results were achieved for plasma coated nanofibers with covalently bonded PRP on the surface.

## 2. Materials and Methods

### 2.1. Preparation of Freeze-Platelet-Rich Plasma 

Platelet-rich plasma (PRP) was prepared as described in [18,33] with minor modifications. Blood was taken from healthy, non-smoking women after obtaining voluntary informed consent. The blood was centrifuged in special tubes (Plasmolifting™, Moscow, Russia) and platelet-rich plasma was collected and activated by a three freeze-thaw cycler. Plasma-derived growth factors were collected by centrifugation at 12,000 *g* for 10 min at 4 °C, and kept frozen at −70 °C until further usage. Each mL of PRP contained 102.5 mg dry matter.

All subjects gave their informed consent for inclusion before they participated in the study. The study was conducted in accordance with the Declaration of Helsinki, and the protocol was approved by the Ethics Committee of the Research Institute of Clinical and Experimental Lymphology–Branch of the Institute of Cytology and Genetics, Siberian Branch of Russian Academy of Sciences (RICEL-branch of ICG SB RAS) (Identifier: N115 from 24 December 2015).

### 2.2. Electrospining of Nanofibers

Nanofibrous used as substrates were prepared by electrospinning of PCL solution. The PCL (80,000 mw, Sigma Aldrich, Darmstadt, Germany) granulated polymer was dissolved in a mixture of acetic acid (99%, Sigma Aldrich, Darmstadt, Germany) and formic acid (98%, Sigma Aldrich, Darmstadt, Germany). The weight ratio of acetic acid to formic acid was 2:1. The concentration of PCL was 9 wt %. The solution was stirred for 24 h at room temperature and then the electrospinning process was carried out using a Nanospider™ NSLAB 500 (ELMARCO, Liberec, Czech Republic). The PCL solution was electrospun with a 20 cm long wired electrode under voltage of 55 kV. The distance between high voltage and ground electrodes was set at 100 mm. The processing of sample is described in our previous works [31]. The as-prepared PCL nanofibers were denoted as PCL-ref.

### 2.3. Deposition and Characterization of Plasma Layers

The deposition of plasma polymers was carried out using a vacuum system UVN-2M equipped with the rotary and oil diffusion pumps providing the residual pressure in a vacuum chamber below 10^−3^ Pa. The capacitively coupled radio-frequency (RF) plasma was driven by a Cito 1310-ACNA-N37A-FF (Comet) RF power supply connected to the RFPG-128 disk generator (Beams & Plasmas, Zelenograd, Russia), 135 mm in diameter, installed into the vacuum chamber. The RF power and the duty cycle was set at 500 W and 5%, respectively. The deposition time was 15 min.

Ar (99.998%), CO_2_ (99.995%), and C_2_H_4_ (99.95%) gases were used as precursors. Gas flow control was carried out using a Multi Gas Contoller647C (MKS, Andover, Massachusetts, USA). Working and residual gas pressures were measured by a VMB-14 unit (Tokamak Company, Petushki, Russia) and D395-90-000 BOC Edwards controllers (Crawley, UK). The distance between RF-electrode and the substrate was set at 8 cm. The flow rates of Ar, CO_2_ and C_2_H_4_ was set to 50, 16.2 and 6.2 sccm, respectively. The plasma coated PCL nanofibers is denoted as PCL-COOH.

The nanostructure of the nanofibers was studied by scanning electron microscopy (SEM) using a JSM F7600 (Jeol Ltd., Tokyo, Japan) device. The SEM micrographs were obtained at an accelerating voltage of 2 kV and a scan time of 1 min. In order to compensate the surface charging, the samples were coated with a ~5 nm thick Pt layer by using magnetron sputtering.

The infrared spectra in the range from 4000–370 cm^−1^ was measured using a Fourier transform infrared (FTIR) spectrophotometer (Bruker Vertex 80V, Ettlingen, Germany) using attenuated total reflectance mode (ATR-FTIR). The data was collected at a pressure of 250 Pa with a resolution of 4 cm^−1^ and 100 scans.

The chemical composition of the sample surfaces was characterized by X-ray photoelectron spectroscopy (XPS) using an Axis Supra (Kratos Analytical, Manchester, UK) spectrometer. The maximum lateral dimension of the analyzed area was 0.7 mm. The spectra were fitted using CasaXPS software (Casa Software Ltd., Teignmouth, UK) after subtracting Shirley-type background. The binding energies (BE) for all carbon environments were employed from the literature [31,34]. The C1s spectra of PCL-ref and PCL-COOH were fitted using the methodology described elsewhere [31,35].

The quantification of COOH groups has been performed by well-known trifluoroethanol (TFE, Sigma Aldrich, Darmstadt, Germany) derivatization and subsequent XPS analysis [36]. The atomic concentration of COOH groups was calculated from the amount of fluorine.

### 2.4. Coating of Scaffolds with PRP

In order to evaluate the adsorption of PRP to as-prepared PCL nanofibers, firstly, the PCL-ref was immersed in PRP solution at room temperature for 15 min and then washed with Phosphate buffered saline (PBS). The sample was denoted as PCL-P1. Afterwards, in order to evaluate the ionic bonding of PRP to plasma coated PCL, nanofibers (PCL-COOH) were immersed in a solution of PRP for 15 min and then washed with PBS (PCL-COOH-P2). Finally, the covalent bonding of PRP to the PCL-PP was performed by subsequent immersing of PCL-COOH in *N*,*N*′-Dicyclohexylcarbodiimide DCC (Sigma Aldrich, 98%, Darmstadt, Germany) solution in water (2 mg/mL) for 15 min. Washing with PBS was followed by incubation with PRP for 15 min at room temperature and final PBS cleaning. These coated scaffolds (denoted as PCL-COOH-P3) were finally placed in 96-well plate. Scaffolds were sterilized under ultra-violet (UV) radiation for 45 min prior to modification by PRP.

### 2.5. Cell Tests

#### 2.5.1. Cell Culture

Human mesenchymal stromal cells were extracted from bone marrow using standard methods [37] and cultured in Dulbecco’s modified Eagle’s Medium: Nutrient Mixture F-12 (DMEM/F12, Sigma Aldrich, Paisley PA4 9RF, UK) that was supplemented with 10% fetal bovine serum (FBS, Gibco, Carlsbad, CA, USA). Cells were seeded in 96-well plates on scaffolds in concentration of 5 × 10^3^ cells/well. The study was approved by the Ethics Committee of the RICEL-branch of ICG SB RAS (No 115 from 24.12.2015).

#### 2.5.2. Cell Attachment

The scaffolds covered with MSCs were fixed using 4% paraformaldehyde solution for 10 min and followed by cell membrane permeabilazation with 0.1% Triton X-100 for 15 min. Then actin filaments of cytoskeleton were stained with Alexa fluor 532 phalloidin (Thermo Fisher Scientific, Carlsbad, CA, USA) for 30 min. Cell nuclei were stained with Hoechst 33342. Finally, the cell adhesion on the scaffold was observed using a fluorescence microscope (Zeiss, Axio observer Z1, Oberkochen, Germany).

#### 2.5.3. Cell Proliferation

The rate of cellular proliferation on the samples of scaffolds was detected using the Click-iT™ EdUAlexa Fluor™ 488 Imaging Kit (Thermo Fisher Scientific, Carlsbad, CA, USA) according to the protocol. EdU is analogue of thymidine and it is efficiently incorporated into DNA during S phase of the cell cycle. Each scaffold was seeded with MSCs adjusted to a population of 5 × 10^3^ cells/well in 96-well plates. The cell-seeded scaffolds were incubated under a humidified atmosphere (5% CO_2_ and 95% air) at 37 °C. For the means of control, MSCs were cultured in 96 well plate under PRP supplemented media at a concentration from 7 to 0.003 vol %, which is equivalent to 7.17 mg^–3^ µg freeze-dried PRP/mL. At 24 and 72 h for MSC EdU was added to each sample for 2 h. Nuclear of cells were counterstained with Hoechst 33342. The cells were observed by means of fluorescence microscopy (Zeiss, Axio observer Z1, Oberkochen Germany) and analyzed using Cell Activision software (Yokogawa Electric Corporation, Tokyo Japan). Cells quantity was obtained from the acquired microscopic images (*n* = 50 each group). The percentage of proliferating cells was calculated as the ratio of EdU-positive cells (green) to the total number of Hoechst-positive cells (blue).

#### 2.5.4. Cell Apoptosis

The DNA binding fluorescent dye Hoechst 33342 was employed in order to assess the morphological changes in apoptosis. Scaffolds cowered with MSCs were stained with Hoechst 33342 for 15 min at room temperature. Later the cells were observed with a fluorescence microscope (Zeiss, Axio observer Z1, Oberkochen, Germany). Apoptotic cells were characterized by the means of morphological alterations, such as condensed and fragmentation of nuclei.

For all results the mean values and associated error standard deviations were calculated. For statistical analysis we have used Statistica 10. The *p* value ≤ 0.05 was considered as statistically significant. In order to evaluate differences between groups, the nonparametric Mann-Whitney U test was used.

## 3. Results

### 3.1. Preparation and Plasma Modification of PCL Nanofibers

**Figure 1:**
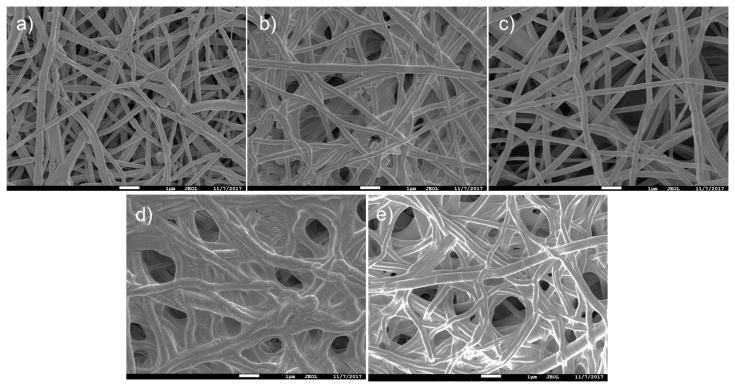
Scanning electron microscope (SEM) micrographs of polycaprolactone nanofibers (PCL-ref) (**a**); PCL-P1 (**b**); PCL-COOH (**c**); PCL-COOH-P2 (**d**) and PCL-COOH-P3 (**e**). The size of the bar corresponds to 1 µm.

**Figure 2:**
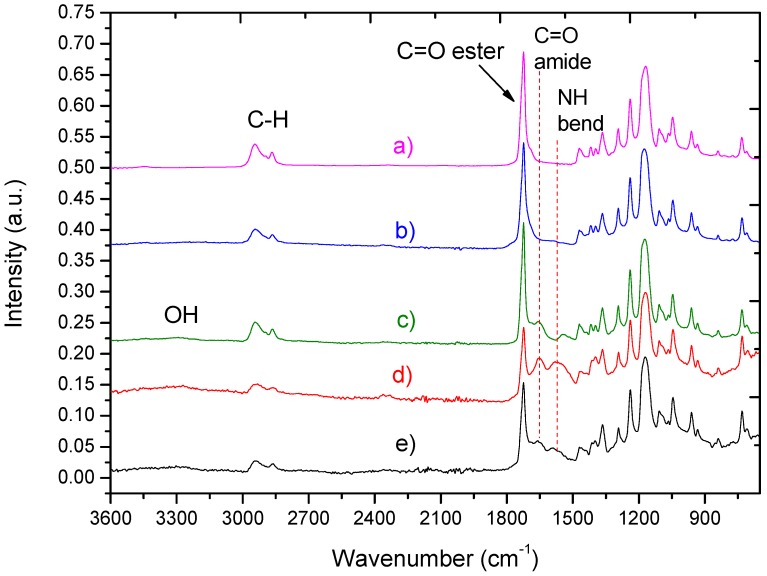
Fourier transform infrared spectrophotometry using attenuated total reflectance mode (ATR-FTIR) spectra of as-prepared nanofibers PCL-ref (**a**); PCL-P1 (**b**); PCL-COOH (**c**); PCL-COOH-P2(**d**) and PCL-COOH-P3 (**e**).

**Figure 3:**
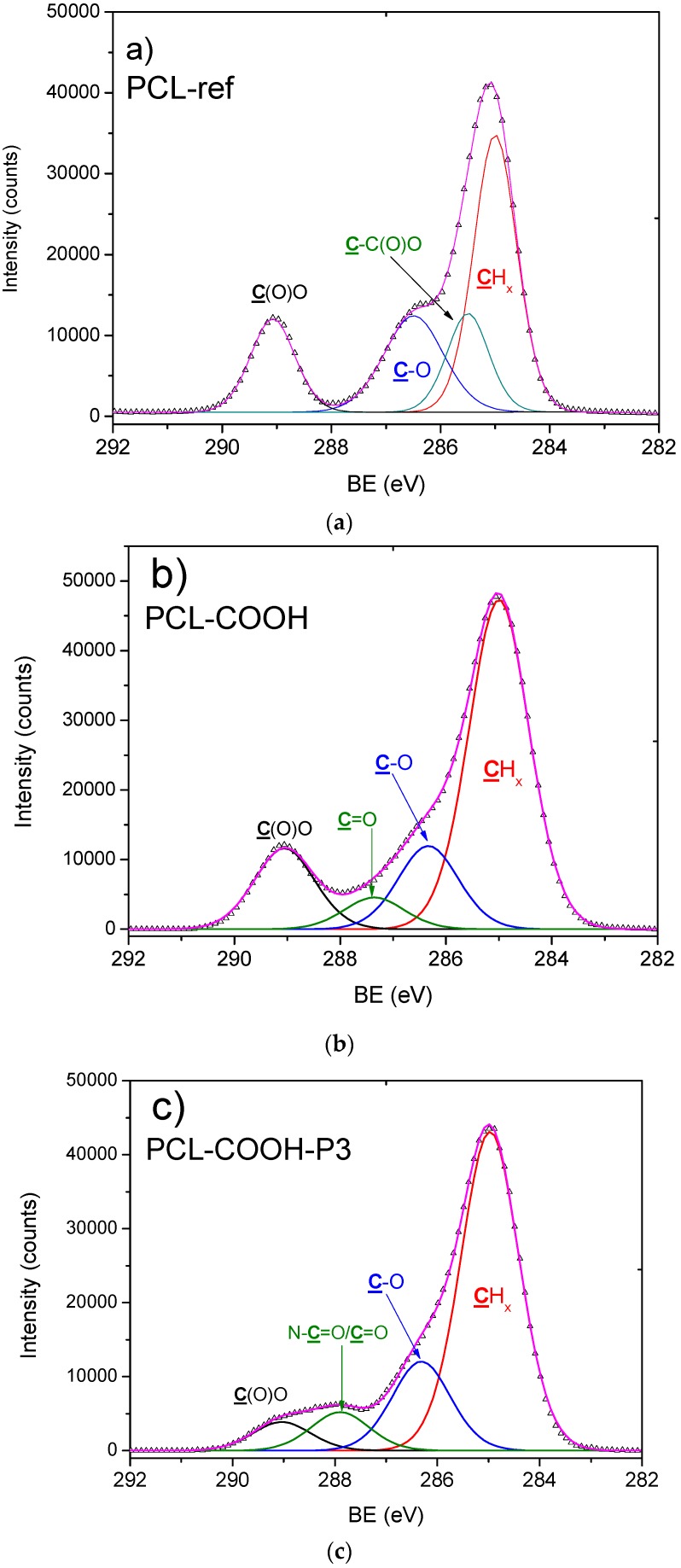
X-ray photoelectron spectroscopy (XPS) C1s spectra of as-prepared nanofibers PCL-ref (**a**); PCL-COOH (**b**) and PCL-COOH-P3 (**c**).

The obtained results indicate that under optimal electrospinning conditions it was possible to prepare homogenous beads-free PCL nanofibers with a diameter of 270 ± 50 nm (Figure 1a). In a previous work, the oxygen/carbon (O/C) ratio of as-prepared PCL nanofibers (PCL-ref) measured by XPS was equal to 0.31, which is slightly lower compared to 0.33 expected from the PCL chemical formula [31]. The high quality of PCL nanofibers was also confirmed by typical ATR-FTIR (Figure 2a) and XPS (Figure 3a) spectra. The C1s XPS spectrum of PCL-ref was fitted using previously reported methodology [31]. The deposition of CO_2_/C_2_H_4_ plasma polymers on the surface of the nanofibers was confirmed by ATR-FTIR (Figure 2b) and XPS (Figure 3c). After comparison of Figure 2a,c, changes in the C=O stretching bands could be observed. XPS revealed that the structure of the C1s curves has been significantly changed after coating by the CO_2_/C_2_H_4_ plasma polymer, and the structure of XPS C1s curve depicted in Figure 3b is similar to the COOH plasma polymers deposited onto Si wafers [38,39]. The concentration of the C(O)O contribution (BE = 289 eV) equals to 11.3 at %, but this carbon contribution includes both esters and carboxyl functions. According to XPS, derivatization density of the COOH groups was performed the derivatization of CO_2_/C_2_H_4_ plasma polymers deposited on Si wafers, as the derivatization of carboxylic groups using the reaction with trifluoroethanol (TFE) is not applicable for PCL nanofibers due to dissolution of PCL in TFE. The derivatization of the COOH groups on the CO_2_/C_2_H_4_ plasma polymers deposited onto Si wafers in the same conditions revealed that this layer bears 0.57 at % of COOH groups. It could be considered that the same amount of COOH groups was deposited on the PCL nanofibers. Regarding the morphology, SEM analysis revealed that it was not influenced by the deposition of the plasma polymerized layer (Figure 1c).

### 3.2. Immobilization of PRP onto PCL Nanofibers

According to ATR-FTIR results presented in Figure 2b, the immersion of PCL-ref in PRP solution has no effect on the layer chemistry of PCL nanofibers, i.e., no PRP immobilization was been demonstrated. On the contrary, the immersion of plasma coated PCL-COOH in PRP solution led to significant changes in the layer chemistry. First of all, it was found that new peaks located at 1652 and 1598 cm^−1^ attributed to C=O stretching and NH_2_ bending of amides, respectively (Figure 2d,e). These new peaks confirm the incorporation of the molecules with peptide bonds.

Moreover, XPS analysis revealed that the PCL-COOH composed of 72.1 at % of carbon and 27.9 at % of oxygen. The immobilization of PRP though ionic bonds (PCL-COOH-P2) led to the incorporation of significant concentration of nitrogen (10.7 at %), whereas the concentration of carbon and oxygen were 70.5 and 18.8 at %, respectively. The immobilization of PRP via covalent bonding (PCL-COOH-P3) led to similar composition of the surface: [C] = 76.5 at %, [O] = 16.3 at % and [N] = 7.2 at %. The XPS C1s signal of PCL-COOH-P3 was fitted using four carbon environments as described elsewhere [10]. The C1s XPS curve fitting revealed significant changes in carbon environment after immobilization of PRP (Figure 3c). The concentration of the C(O)O environment decreased while new contribution corresponding to the amides (N–C=O, BE = 288 eV) appeared. This observation confirms that the biomolecules with protein nature was grafted onto the surface. It is worth noting that according to SEM micrographs, the PCL-P1 exhibited same morphology as PCL-ref (Figure 1a,b). In contrast, the immobilization of PRP on PCL-COOH led to significant modification of PCL nanofibers morphology. Irrespective of the nature of bonding (ionic or covalent), the pores of nanofibrous mats became smaller due to the immobilization of PRP. Nevertheless, both PCL-COOH-P2 and PCL-COOH-P3 exhibited nanofibrous structures similar to ECM.

### 3.3. Cell Adhesion Test

The first stage of the biocompatibility evaluation of the scaffolds was the investigation of the cell adhesion to the surface of the prepared PCL-ref and modified nanofibers PCL-COOH, PCL-COOH-P2 and PCL-COOH-P3.

**Figure 4:**
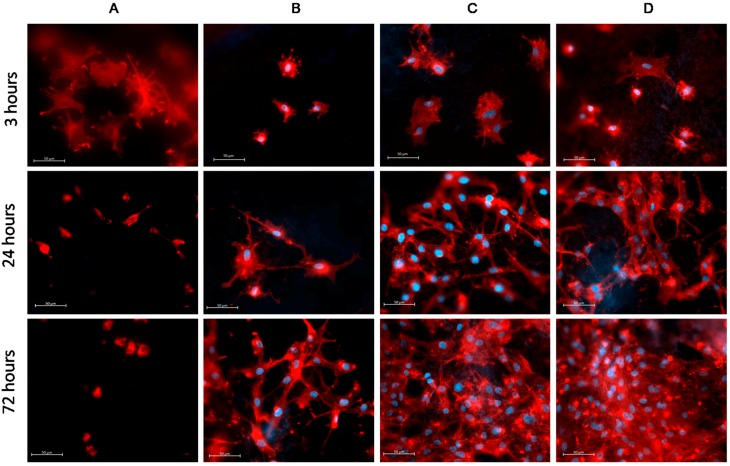
Adhesion of mesenchymal stromal cells (MSCs) on the surface of PCL-ref (**A**); PCL-COOH (**B**); PCL-COOH-P2 (**C**) and PCL-COOH-P3 (**D**). The actin filaments of cytoskeleton are stained by Phalloidin (red) while the cell nucleus is stained by Hoechst 33342 (blue). All images shown at ×40 magnification and the size of the bar corresponds to 50 µm.

It was found that after 3 h, the MSCs seeded on PCL-ref exhibited weak coloring of the cytoskeleton. The adhesion contacts of cells with the surface of the PCL-ref was very weak. Cells attempted to adhere to the hydrophobic surface, leading to the formation of the lamellipodia and actin-rich filopodia (Figure 4). However, the absence of a good contact with the PCL-ref surface affected the survival and proliferation of MSCs, and the majority of cells did not survive on PCL-ref after 72 h.

The adhesion of MSC cells to the PCL-COOH and PCL-COOH-P2 was characterized by smaller spreading area, but the cells formed adhesion contacts. The cells had significantly well-defined network of actin filaments (Figure 4). It has been observed that the MSCs on PCL-COOH-P3 exhibited very good spreading and very meaningful actin-rich contacts with the surface as well as defined network of stress-fibrils. It is worth noting that the Hoechst/Phalloidin staining of the PCL-ref led to the very noisy background on the picture due to interaction of the dye with the hydrophobic PCL nanofibers. Thus, in order to provide better quality images, the cells on PCL-ref were stained with Phalloidin only.

Hence, it was shown that the adhesion of MSCs to the modified surfaces (PCL-COOH, PCL-COOH-P2 and PCL-COOH-P3) resulted in the formation of the significant actin-rich cytoskeleton, which is an important compound for cell viability and fitness. It is well-known that PRP contains adhesive protein-fibronectin, and this protein enhances cell adhesion, migration, and proliferation. Moreover, fibronectin participate in the control of cell differentiation, and cytoskeleton sustainability [16].

The best cell adhesion results were observed for PCL-COOH-P2 and PCL-COOH-P3, which can be explained by a high amount of PRP fibronectin at its surface.

### 3.4. Influence of the Surface Modification on the Proliferation of MSCs

**Figure 5:**
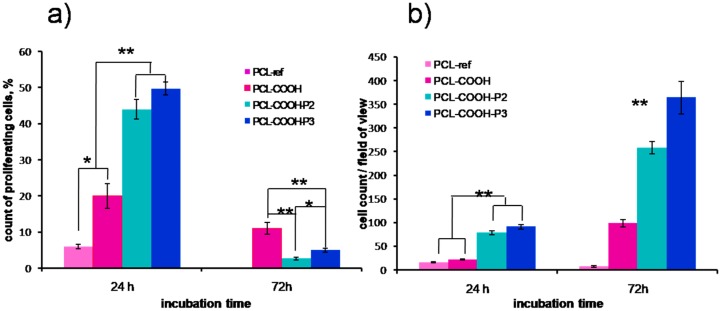
Influence of surface modification of PCL nanofibers on proliferation and viability of human MSCs. (**a**) the percentage of proliferating cells after 24 and 72 h of cultivation (calculated as the ratio of EdU-positive cells to the total number of Hoechst-positive cells); (**b**) average number of cells for single bright-field image after 24 and 72 h of cultivation. Data are expressed as mean ± standard deviation. ** *p* < 0.01, ** *p* < 0.05.

**Figure 6:**
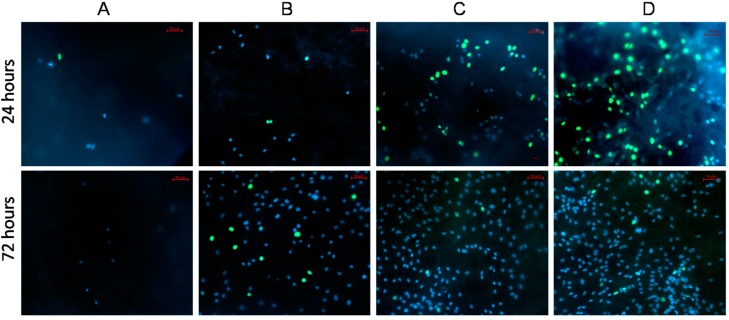
Influence of surface modification of PCL-ref (**A**); PCL-COOH (**B**); PCL-COOH-P2 (**C**) and PCL-COOH-P3 (**D**) nanofibers on proliferation and viability of human MSCs. The cell nucleus is stained by DNA binding fluorescent dye Hoechst 33342 (blue), the proliferating cells stained by EdUAlexa Fluor™ 488 (green). All images shown at ×20 magnification.

The influence of PRP immobilization on the proliferation of the adhered human MSCs is demonstrated in Figure 5 and Figure 6. After 24 h, the highest percentage of proliferating cells (equal to 50 ± 1.7%) has been recorded for PCL-COOH-P3, where the PRP biomolecules have covalent bonding to the scaffold surface. Control cells were cultivated on plastic plates in media supplemented with PRP in range concentration from 7 to 0.8 vol % demonstrated the proliferation rate comparable PCL-COOH-P3 (51.8%–49.7%).

The proliferation on PCL-COOH-P2, where biomolecules have ionic bonding with the surface, was also very high and the percentage of proliferating cells was equal to 44 ± 2.7%. At the same time, the percentage of proliferating cells on PCL-COOH and PCL-ref was only 20 ± 3.4% and 6 ± 0.8%, respectively. The good adhesion and active cell proliferation led to high average number of cells counted from a single bright-field image (91 ± 4.8 cells for PCL-COOH-P3 and 78 ± 4.2 for PCL-COOH-P2), while the number of cells on PCL-COOH and PCL-ref was lower (22 ± 1.8 and 16 ± 0.9, respectively).

Nevertheless, remarkably high level of proliferation on PCL-COOH-P3 occurred after 24 h, and the proliferation slowed down after 72 h, due to contact inhibition (normal cells stop growing when they reach confluence) (Figure 5 and Figure 6). On PCL-COOH, the density of the cell culture was lower and the proliferation level after 72 h was the highest (11%). Cells on PCL-ref scaffold stopped dividing after 72 h. These samples exhibited very high average number of cells: 258 ± 12.9 and 364 ± 34.5 for PCL-COOH-P2 and PCL-COOH-P3, respectively, as a result of active proliferation and sustaining the cell viability on nanofibers with immobilized PRP. The average cell number on PCL-COOH was only 98 ± 8, whereas PCL-ref exhibited only single viable cells (Figure 5 and Figure 6).

### 3.5. Influence of The Surface Modification on the Viability of MSCs

**Figure 7:**
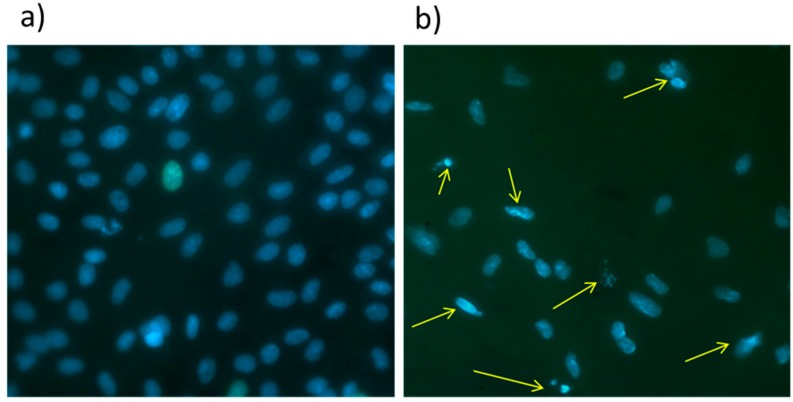
Representative picture of apoptosis analysis. The morphology of MSCs nucleus after 24 h. The PCL-COOH-P3 (**a**) exhibited homogenous form and coloration of the nucleus and no condensation of the chromatin; whereas cells grown on PCL-ref (**b**) exhibited the chromatin condensation and nuclear fragmentation (kariorhexis) (yellow arrows). Magnification ×40.

The percentage of apoptotic cells was determined by staining of the cell nucleus by Hoechst 33342. It was found that after 24 h, 23 ± 5% nuclei adhered to PCL-ref and showed some features of apoptosis, e.g., fragmentation of the nucleus and condensation of chromatin, which is shown in Figure 6. In contrast, the modified PCL nanofibers exhibited low percentages of apoptotic/death cells (1 ± 0.06%) (Figure 7).

After 72 h the number of apoptotic cells on PCL-COOH-P2 and PCL-COOH-P3 did not increase in spite of full confluence of cells. At the same time, the percentages of apoptotic cells on PCL-ref exceeded 50%.

It could be concluded that the deposition of plasma layers combined with immobilization of PRP enhances the viability and proliferation of human MSCs. The covalent binding of PRP leads to extension of this effect compared to the ionic bonding of PRP. The PCL-COOH-P3 exhibited the total cell number 1.5 times higher compared to PCL-COOH-P2.

Therefore, the immobilization of PRP onto PCL nanofibers leads to a significant increase in the number of adhered cells, and an increase of the density of the cell population and proliferation level, and a decrease in apoptotic/dead cells.

However, the ionic bonding of PRP to PCL-COOH demonstrated less effect on proliferation level enhancement although XPS analysis of PCL-COOH-P2 and PCL-COOH-P3 after 72 h of immersion in water revealed the same composition of the surface (within XPS error margins), i.e., the bonding of proteins, even via ionic linkage is strong. The influence of the nature of the linkage between the PRP and PCL-COOH will to be further studied in future using mass spectra analysis (e.g., by Matrix Assisted Laser Ablation Time-of-Flight mass spectrometry).

High proliferation rate, low percentage of apoptotic cells death, contact inhibition of proliferation MSCs on substrates with PRP suggests that the investigated scaffold provides conditions for normal cell function. Immobilization of the PRP on plasma coated PCL nanofibers used in this investigation is a promising technique to modification of surface properties for enhancement of biological activity of tissue engineering scaffolds.

Thus we have shown developed a functional ECM mimicking scaffold that is capable of consolidating platelet-derived growth factors thereby to supply adhesion and high proliferation of MSCs.

## 4. Conclusions

The immobilization of platelet-rich plasma (PRP) onto plasma coated polycaprolactone (PCL) nanofibers significantly improves cell adhesion, boosts cell proliferation and viability. It was shown that the plasma coating solely can enhance the biocompatibility of the PCL nanofibers, but in this case the level of cell proliferation is dramatically lower compared to the PCL nanofibers with immobilized PRP. The immobilization of the PRP to the plasma coated PCL-COOH can be easily used by soaking in a solution of PRP. Our results have shown that the bonding of PRP (ionic or covalent) influences the cell proliferation level and cell viability. The in vitro tests of plasma-coated PCL nanofibers with covalently immobilized PRP showed best performance, and this material is highly promising for tissue engineering.
